# Is Unidirectional Drying in a Round Capillary Always Diffusive?

**DOI:** 10.1021/acs.langmuir.3c00169

**Published:** 2023-04-06

**Authors:** Romane Le Dizès Castell, Marc Prat, Sara Jabbari Farouji, Noushine Shahidzadeh

**Affiliations:** † Institute of Physics, University of Amsterdam, 1098 XH Amsterdam, The Netherlands; ‡ Institut de Mécanique des Fluides de Toulouse (IMFT), Université de Toulouse, 31400 Toulouse, France

## Abstract

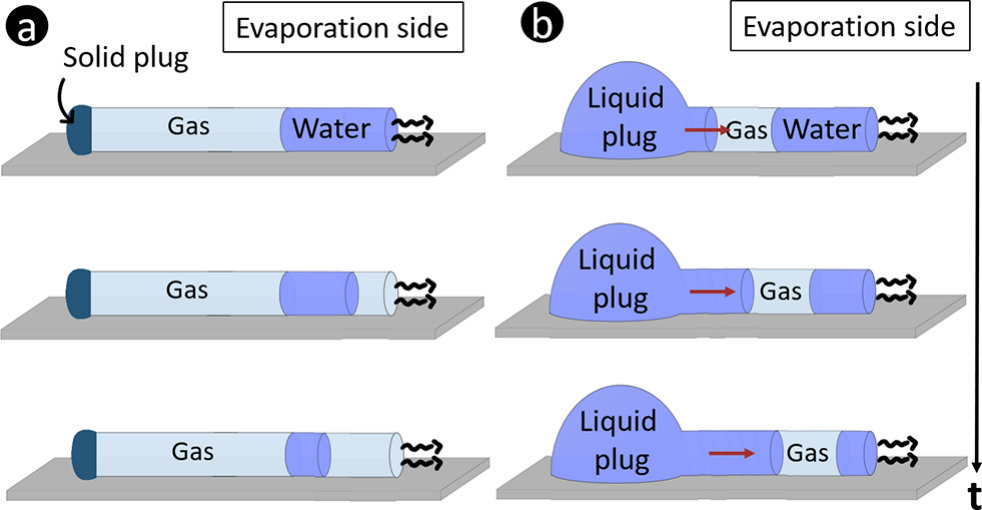

The unidirectional drying of water in cylindrical capillaries has been described since the discovery of Stefan’s solution as a vapor diffusion-controlled process with a square root of time kinetics. Here we show that this well-known process actually depends on the way the capillary is closed. Experiments are performed on the evaporation of water in capillaries closed at one end with a solid material or connected to a fluid reservoir. While we recover Stefan’s solution in the first case, we show that in the second situation the water plug evaporates at a constant rate with the water–air meniscus remaining pinned at the exit where evaporation proceeds. The presence of the liquid reservoir closing the capillary combined with a capillary pumping effect induces a flow of the water plug toward the evaporation front leading to a constant-rate drying, substantially faster than the prediction of Stefan’s equation. Our results show that a transition from a constant-rate evaporation regime at short times to a diffusion-driven evaporation regime at long times can be observed by increasing the viscosity of the fluid in the reservoir blocking the other end of the capillary. Such transition can also be observed by connecting the capillary end to a solidifying fluid like epoxy glue.

## Introduction

Understanding the drying processes of porous media is of major importance for soils in agriculture,^1^ civil engineering, petrophysics, or the conservation of stone artworks^2^ and has been the subject of many studies. One can refer to ref (3) for a recent review of literature with numerous references. However, the accurate prediction of the evaporation rate from a porous medium remains a challenge.^3,4^ Modeling of flow through porous materials is often based on the concept of considering the pore network as a bundle of parallel cylindrical tubes with varying diameters.^5,6^ A capillary can also be considered as the elementary unit in pore network models,^7^ an approach that has proven to be an effective way to gain meaningful insights into the transport and drying properties of porous media.^8^ In other words, the consideration of a single capillary is both interesting for highlighting the physics at the pore scale and in the prospect of scale bridging via pore network or lattice Boltzmann models,^9^ for instance. Stefan^10^ in 1871 was the first to describe the diffusion-driven drying in a vertical capillary tube: he showed that the evaporation of a liquid in a circular tube slows down as the air–liquid interface is receding inside the tube.^11^ Since then, round capillaries have been used in several studies to understand the flow and transport processes with different fluids such as ionic solutions^12,13^ or colloidal suspensions.^14^ Unidirectional drying in capillaries has also been extensively studied by changing different parameters including geometry and wettability properties of capillaries.^15−17^ Generally, capillaries of polygonal cross section are considered to be closer to the geometry of real pores due to the presence of corners and angles where thick capillary films can form and strongly influence the drying kinetics^15,18,19^ or imbibition process.^20^ Nevertheless, most of the previous studies focus on unidirectional drying in fully saturated capillaries and closed by a solid phase (glass or epoxy glue, for instance). In nature, however, drying of a porous medium can happen in a lot of different configurations. For instance, during evaporation, the initially liquid-filled pores are gradually occupied by gas: this gas invasion leads to disconnections in the liquid pathway to the surface, thus creating discontinuous water flows.^17^ Drying can therefore happen in dead-end and two open-end pores but also in pores connected to a supply in water. It can be also noticed that the consideration of tubes or channels with two open ends is classical in the studies of drying of colloidal suspensions.^14,21,22^

Here we show that the classical description of unidirectional drying in capillaries used as model pores depends on the closing condition of the capillary. While drying in cylindrical capillaries closed by a solid plug follows a classical diffusion-driven behavior, a faster capillary-drying regime, characterized by a constant-rate period,^23,24^ can be induced when the capillary end is in contact with a liquid plug. Although this case is similar to the constant-rate period (CRP) observed within porous media,^23,24^ drying of a single capillary cannot be representative of all the mechanisms explaining the CRP observed with porous media.^3^ In the case of porous media, the CRP is notably due to the fact that many pores remain saturated at the surface as the result of the preferential invasion of the coarser pores. In the case of a single tube, the CRP is obtained because the meniscus stays pinned at the tube end, somewhat similar to the situation of finer pores in a porous medium.

More generally, the present study can be seen as a contribution to the current trend of addressing drying in situations of greater complexity^12−14,20−22^ than the classical drying problems with a pure liquid. The presence of a gas plug trapped in a tube and the presence of two different liquids in the tube during drying are new features of our study. In relation to the present study, it can be also noted that the formation of trapped gas plugs is likely under vacuum drying conditions.^25^ Although the experiments presented in the Article are not performed under vacuum conditions, a gas plug is present.

## Experimental Section

**Figure 1:**
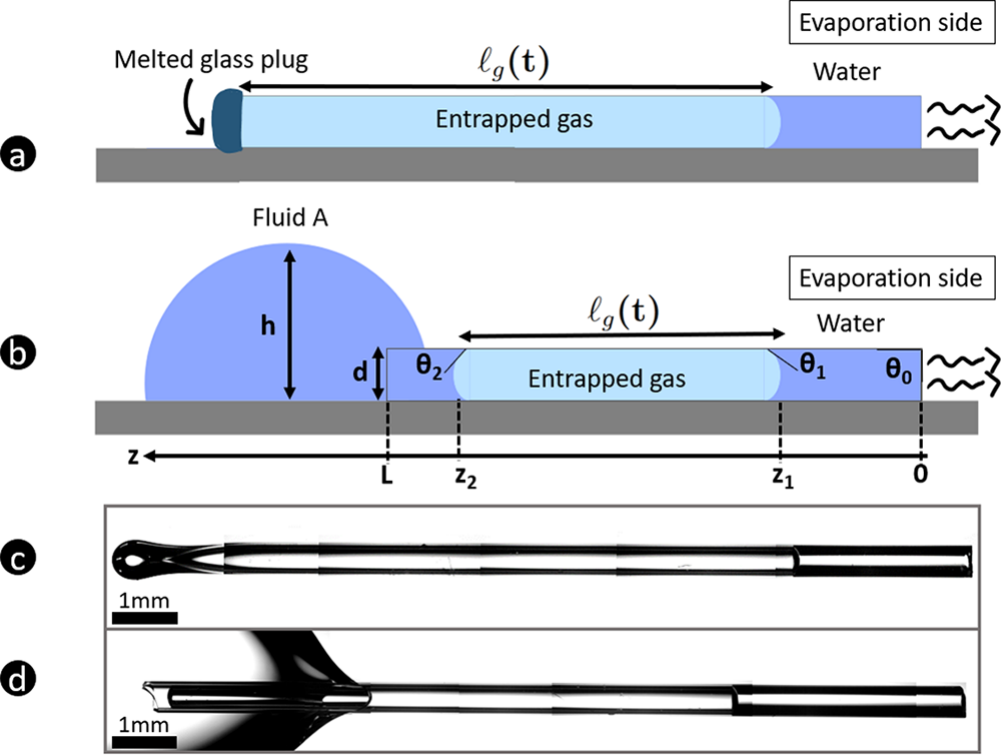
Schematics and pictures of unidirectional drying in round capillaries for the two studied configurations. In configuration a, the left end of the capillary is melted whereas in configuration b the capillary is closed by a liquid droplet deposited at the left end. Panels c and d are microscopy images of configurations a and b at the start of the experiment, *t* = 0. The evaporating water corresponds to the blue-shaded zone on the right side of the images.

We used in this study round and square borosilicate glass capillaries purchased from VitroCom, cut to a length of roughly 20 mm (of inner diameter *d* = 0.5 mm and wall thickness *w* = 0.1 mm for round capillaries and of width 0.5 mm, wall thickness *w* = 0.1 mm for square capillaries). The capillaries are cleaned with ethanol and demineralized water and dried for 24 h at 60 °C. The unidirectional drying is subsequently investigated in two situations as illustrated in Figure 1a and b.

### a Capillaries Closed by a Solid Plug

One end of the capillary is melted with a torch prior to the filling with water. Once melted, an ultrathin Pasteur pipet is used to partially fill the capillary with demineralized water (*V*_0_ ≈ 0.6 μL). A gas plug is therefore present in between the melted end and the entrapped volume of water evaporating.

### b Capillaries Connected to a Liquid Plug

The capillary is partially filled with demineralized water (by capillary suction, *V*_0_ ≈ 0.6–1 μL) and placed horizontally. A big droplet of liquid (roughly 2 mL) is deposited at the other end of the capillary opposite to the entrapped volume of water evaporating. Here again, some air remains trapped inside the capillary between the entrapped water evaporating from the open side and the large droplet of liquid (here defined as fluid A) closing the other end of the capillary. The volume of the latter is maintained roughly constant during the experiment. The impact of the viscosity of fluid A on the kinetics of the drying of water plugs is investigated by using different liquids as fluid A: demineralized water, glycerol (Sigma-Aldrich), and PDMS 100000 cSt (Sigma-Aldrich). Finally, in some experiments, nail polish purchased from Etos and epoxy glue purchased from Liquimoly are used as fluid A to close the capillaries.

These two configurations can be seen as pore scale configurations corresponding to the drying situation (configuration a) and the evaporation-wicking situation (configuration b) in porous media studies.^26^

All the experiments are performed horizontally to avoid the effect of gravity. The evaporation of the entrapped water inside the capillary is monitored using an inverted Leica DM IRM microscope with a 2.5 magnification objective for both configurations. An image is taken every 10 s until the end of the drying. The relative humidity is measured for each experiment using a Testo 645 thermo-hygrometer. The temperature is kept at 20 °C.

## Results and Discussion

**Figure 2:**
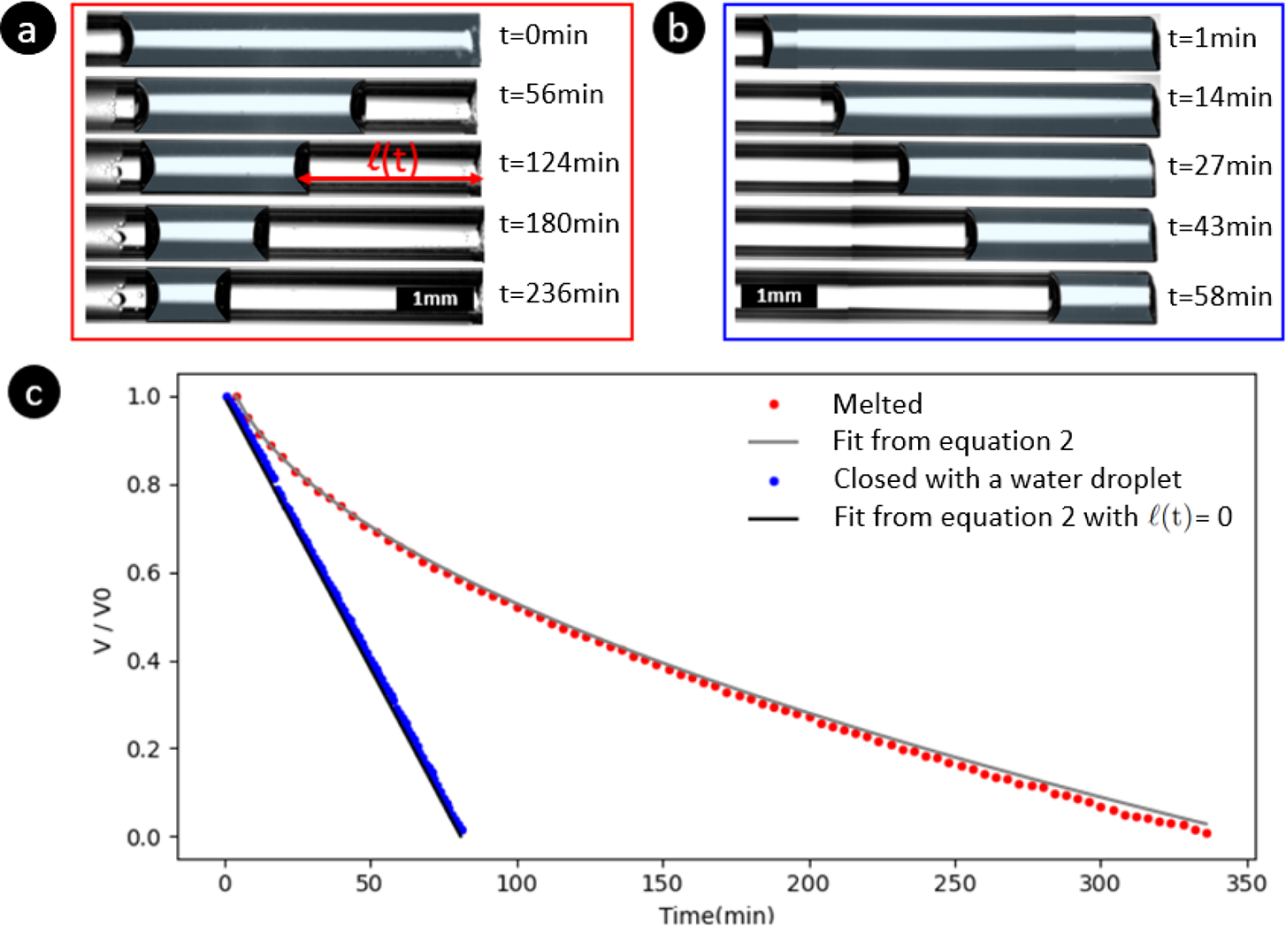
Microscope pictures of the drying of water (blue-shaded zone in the pictures) in cylindrical capillaries with a melted end (a) and closed by a water droplet (b). In both configurations, the water is evaporating from the right. (c) Volume of the water plug in the capillary divided by the initial volume as a function of time (minutes). The red dots are experimental results for water evaporating in melted-end capillaries whereas the blue dots are the experimental results for capillaries closed by a water droplet. For the sake of clarity, only one example of each situation is plotted in the figure, but four experiments have been performed for each situation.

The results for the two configurations are shown in Figure 2, where the ratio of the volume of entrapped water at the time *t* relative to its initial value is plotted as a function of time. Surprisingly, we observe two different drying behaviors for the capillaries with a melted end (configuration a) and those connected to a liquid reservoir (configuration b). In situation a, where one end of the capillary is melted (solid plug), the water meniscus on the evaporation side of the capillary starts receding inside the capillary immediately. The water meniscus on the closed side of the capillary (position *z*_1_ in Figure 1) remains in place and does not move during drying. Consequently, the volume of the gas plug *V*_g_ (entrapped gas in Figure 1) remains constant. On the contrary, in configuration b, the water meniscus on the evaporation side remains almost pinned at the exit during the drying, and the evaporation rate is constant. Water thus evaporates substantially faster compared to situation a.

In case a, as the bulk meniscus is receding inside the capillary, the evaporation rate decreases continuously because the vapor has to diffuse over an increasing distance inside the capillary in agreement with previous reports in the literature on Stefan’s tube problem.^10,11^ The evaporation rate  in kg/s can be expressed from Fick’s law:^27^
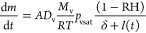
1where *m* = *Aρ*_w_(*t*) is the evaporated mass and (*t*) is the distance between the water–air meniscus on the evaporation side and the exit of the tube, see Figure 2a. *A* is the cross-sectional surface area of the capillary, ρ_w_ is the density of liquid water, *D*_v_ is the vapor molecular diffusion coefficient (*D*_v_ = 0.25 × 10^–4^ m^2^/s^28^), *R* is the universal gas constant, *M*_v_ is the vapor molecular weight, *T* is the temperature, RH is the relative humidity of the ambiance, and *p*_vsat_ is the saturated vapor pressure at the considered temperature (*p*_vsat_ = 2.3 kPa). δ is defined as the vapor external transport length characterizing the vapor diffusive transport between the open end of the tube and the external air.^19^ Our experiments differ from Stefan’s situation due to the entrapped gas plug in the capillary. Nevertheless, as its volume remains constant, eq 1 is still valid. In this case, (*t*) can be obtained by equating the evaporation rate from eq 1 with . Integrating the evaporation rate with the initial condition (*t* = 0) = 0 leads to the following equation for the evolution of the position of the air/water meniscus on the evaporation side of the capillary (right meniscus in Figure 2a):
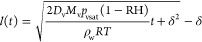
2Using *V*(*t*) = *V*_0_ – *A* × (*t*) (where *V*_0_ is the volume of water in the capillary at *t* = 0), we can predict the time evolution of liquid volume *V* and compare it with the experimental results shown by the solid line in Figure 2c. δ is computed from eq 2 for every experiment performed and is found to be equal to 0.30 ± 0.06 mm. This value is in line with the result reported in ref (15) where it is shown that δ is equal to 0.77*d* for a square tube, where *d* is the tube internal side length (in our case 0.77*d* = 0.385 mm ). Thus, the experimental data are in good agreement with the theoretical prediction of the Stefan model demonstrating that the evaporation rate is controlled by diffusion and is not affected by the entrapped gas plug.

**Figure 3:**
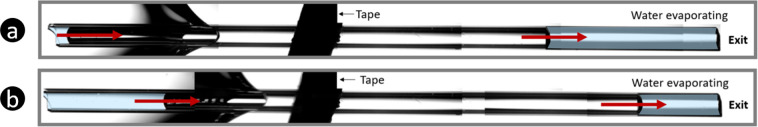
Microscope pictures of the full capillary of inner diameter 0.5 mm closed by a water droplet (right side) at *t* = 0 min (a) and *t* = 43 min (b). The blue-shaded zones show the water evaporating from the right side and entering the capillary from the droplet on the left side.

Unexpectedly, in case b, the volume of the water plug drops linearly with time, and the evaporation rate is constant. Moreover, performing the experiments with different geometries (round or square) does not affect the evaporation rate in contrast to the dead-end pore situation where the geometry can affect the receding meniscus and induce a much faster evaporation due to the liquid film flows in the corners of the square capillary^15,29^ (see Supporting Information for the experimental results with square capillaries). Experimentally, we can notice that fluid A enters the capillary (visible in Figure 3) at the same volumetric rate as the evaporation rate. The volume of the gas plug inside the capillary remains thus constant during the whole drying. In order to keep the water meniscus pinned at the exit during the whole evaporation period, water must flow toward the exit of the capillary as the evaporation proceeds. It could be assumed that this flow results from the spontaneous imbibition of fluid A into the tube according to the well-known capillary suction mechanism described by Washburn.^30^ However, as discussed in some detail in the Supporting Information, the spontaneous imbibition step is very fast (less than 1 s for all the fluids tested) and stops very rapidly due the pressure buildup in the entrapped gas plug until a quasi-hydrostatic distribution of the fluids in the capillary is reached.^31^ Evaporation is negligible during this fast transient step, and the fluid A penetration distance into the capillary during the spontaneous imbibition step is quite limited (a few tens of microns according to the results reported in the Supporting Information). The hydrostatic equilibrium condition is that the pressure *P*_Aeq_ is uniform in fluid A entering the capillary. Thus, from Young – Laplace equation, the capillary pressure in fluid A is
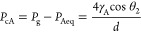
3where γ_A_ is the fluid A surface tension and *P*_g_ is the pressure in the gas plug. For a tube of 0.5 mm in diameter and water as fluid A (θ_2_ ≈ 40°), the overpressure in the tube is 450 Pa. We can verify if this is correct by determining the length of the gas plug from the ideal gas law:
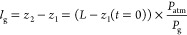
4In Figure 3, *z*_1_(*t* = 0) ≈ 5 mm and *L* = 19.4 mm, the length of the capillary. This yields  = 14.3 mm according to eq 4, which is in very good agreement with Figure 1, where  ≈ 14.2 mm. Note that we have considered for this calculation that *P*_Aeq_ ≈ *P*_atm_. In fact, the pressure in fluid A at the entrance of the tube is slightly greater due to the weight of the liquid in the droplet above the tube entrance. The height of the droplet *h* is limited by gravity effect and is given by , where  is the capillary length^32^ ( = 2.7 mm for water). The corresponding additional hydrostatic pressure at the entrance of the tube can be estimated as . For θ_2_ = 40° for instance, this gives *P*_Aeq_ – *P*_atm_ = 18 Pa, which is small compared to the capillary pressure (*P*_cA_ = 450 Pa). Considering this correction does not change the previous estimate,  = 14.3 mm.

The conclusion is therefore that the system operates with a quasi-static distribution of the fluids in the capillary in the long evaporation step that follows the very fast and limited imbibition step. Nevertheless, a flow is induced in the entrapped water evaporating the gas plug and fluid A (liquid plug) since the gas plug moves in direction of the pinned evaporative meniscus. This flow in entrapped water for round capillaries can be expressed using Poiseuille’s law as
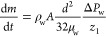
5where μ_w_ is the water dynamic viscosity. Applying this relationship at the beginning of the evaporation step, i.e., for *z*_1_ = 5 mm, yields Δ*P*_w_ ≈ 10^–3^ Pa, which is completely negligible compared to the capillary pressure (450 Pa). The pressure difference Δ*P*_w_ is the viscous pressure drop in the evaporating water plug resulting from the evaporation process. As a result, the pressure in this water plug is slightly lower at the evaporating meniscus than at the left meniscus (Figure 3). This implies that the meniscus curvature is less at the evaporating meniscus (roughly the liquid pressure in this water plug is *P*_w-left_ ≈ *P*_atm_ at the left meniscus whereas it is *P*_w-right_ ≈ *P*_atm_ – Δ*P*_w_ at the right meniscus). Since Δ*P*_w_ is quite small compared to the maximum capillary pressure (450 Pa), the evaporating (right) meniscus is in fact almost flat, whereas the left meniscus is at maximum curvature (this is qualitatively visible in Figure 3). Similarly, the fluid A volumetric flow rate *q*_v_ can be expressed as . For *z*_2_ = *L* – 5 mm (at the end of drying), this gives for water as fluid A a similar negligible pressure drop Δ*P*_A_. The viscous pressure drop in the gas plug is even more negligible due to the much lower viscosity of air.

To evaluate the influence of fluid A viscosity on the drying regime of water, glycerol (μ = 1.4 Pa·s) and PDMS (μ = 98 Pa·s ) are used as liquid reservoirs. Experimentally, the viscosity of fluid A does not seem to impact the drying kinetics: the water–air meniscus remains pinned at the exit of the capillary, and drying proceeds with the same constant rate. However, the estimate of the pressure drop in fluid A toward the end of drying, i.e.,
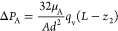
6for the most viscous fluid considered in the experiment (PDMS, μ_A_ = 98 Pa·s) and for *L* – *z*_2_ = 5 mm, gives Δ*P*_A_ = 96 Pa, which is comparable to the capillary pressure ( Pa for PDMS with γ_A_ = 20 N·m^–1^^33^). Thus, contrary to water for which the pressure variation in the gas plug is negligible, the pressure in the gas plug *P*_g_ decreases during the evaporation process when fluid A is very viscous, varying from *P*_atm_ + *P*_cA_ to *P*_atm_ + *P*_cA_ – Δ*P*_A_.

Interestingly, it can be anticipated that if a larger initial volume of entrapped water evaporates in the capillary, a transition from a constant evaporation rate period to a diffusive evaporation period could be observed. For such a transition to occur, the pressure in fluid A plug must become lower than the pressure *P*_atm_ – *P*_cw_ at the air/water meniscus located for *z* = 0 (Figure 1). Neglecting the viscous pressure drop in the water plug, the pressure in the gas plug is given by *P*_g_ – *P*_cw_ = *P*_atm_ – *P*_cw_, which corresponds to Δ*P*_A_ = *P*_cpdms_. Solving eq 6 with Δ*P*_A_ = *P*_cpdms_ gives the distance *L* – *z*_2t_ over which the fluid A meniscus must flow in the tube before a transition from a constant evaporation rate period to a falling evaporation rate period could be observed. We introduced here *z*_2t_, the length *z*_2_ for which the transition between the two regimes occurs. This gives *L* – *z*_2t_ = *Ca*^–1^*d*, with *d* the capillary diameter and , where the evaporation velocity is given by . The dimensionless number *Ca* is the capillary number characterizing the relative effects of viscous forces versus capillary forces. For the PDMS experiment, *Ca*^–1^ ≈ 16. Thus, in the case of the experiment with PDMS, *L* – *z*_2*t*_ = 16*d* ≈ 8 mm. For the studied configuration, an initial water plug greater than about 8 mm should therefore be sufficient to observe the transition. In the case of porous media, it is well established that viscous effects induce the formation of a receding evaporation front in the material.^8^ Although the situation is somewhat more complex in the presence of two liquids of different viscosity separated by a gas plug, the simple configuration studied here indicates that a similar transition can be observed in a simple model pore by playing with the viscosity of fluid A acting as the liquid plug.

**Figure 4:**
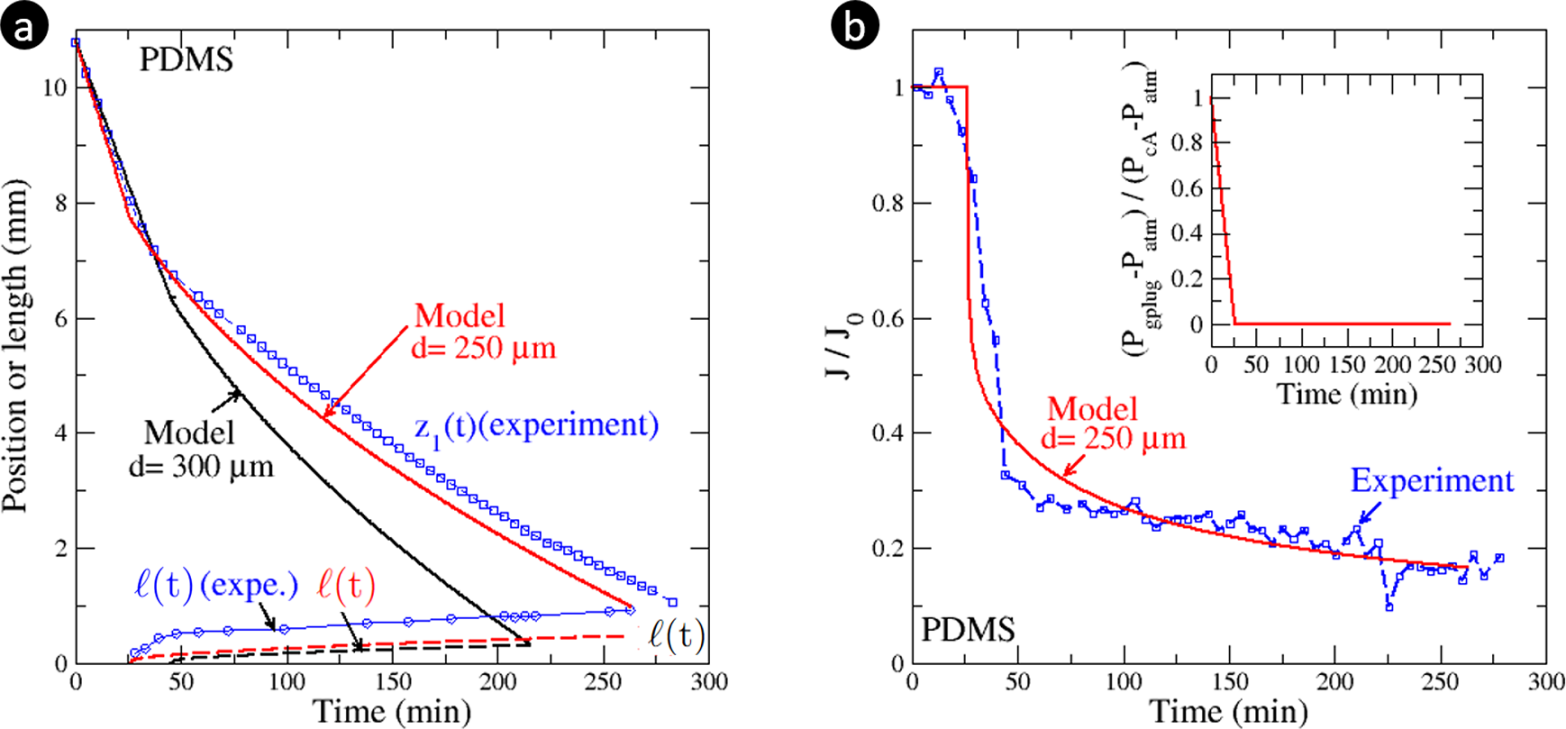
(a) Impact of fluid A penetration on the drying kinetics for configuration b of Figure 1. Refer to Figure 1 for the definition of *z*_1_ and *z*_2_ and to Figure 2a for the definition of . (b) Drying kinetics ( is the evaporation rate; *J*_0_ is the evaporation rate when the evaporative meniscus is pinned at the evaporation exit). The inset shows the pressure variation in the gas plug computed from the model in the Supporting Information. The experimental results are plotted in blue whereas the results obtained with the model are shown in red for capillaries of *d* = 250 μm and in black for capillaries of *d* = 300 μm.

**Figure 5:**
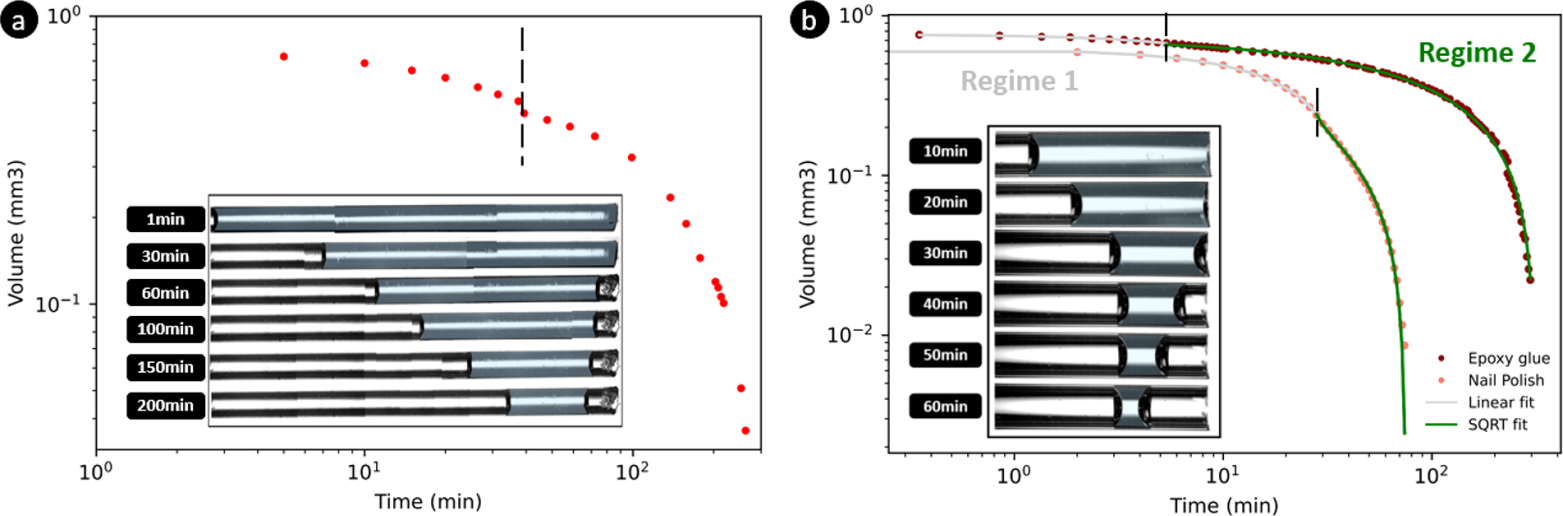
Volume of entrapped water evaporating in a cylindrical capillary plotted as a function of time. In (a), the capillary (*d* = 0.3 mm) is closed from the left side with PDMS, and the two evaporative regimes can be clearly identified. In (b), the capillary (*d* = 0.5 mm) is closed with a drop of nail polish or of epoxy glue: the change in the drying kinetics of water indicates the phase transition of the nail polish and epoxy glue from their liquid states to their solidlike states. The microscope pictures show the drying of water in the capillary closed by a droplet of nail polish.

As described in the Supporting Information, the impact of the viscosity can be considered in a model aimed at predicting the drying kinetics. This model indicates that the transition can be also expected by reducing the tube diameter instead of modifying the initial length of the water plug, keeping all other parameters the same. This results from the fact that the viscous pressure drop in the PDMS plug varies as *d*^–2^. Reducing the tube diameter also has an impact on the evaporation velocity since δ ∝ *d*. Taking into account this impact, by reducing the tube diameter from 500 to 300 μm, one obtains *Ca*^–1^ ≈ 9.6. Thus, *L* – *z*_2*t*_ ≈ 9.6*d* = 2.88 mm, and an initial plug shorter than the ones in the previous experiments should be sufficient to observe the transition between the two evaporative regimes with a tube of 300 μm in diameter. To confirm the relevance of this model and the associated predictions, an experiment with a longer capillary of length *L* = 31.6 mm and diameter 300 μm is performed with a longer initial water plug (*z*_1_(*t* = 0) = 10.8 mm). This leads to the results depicted in Figure 4 and Figure 5a. As expected, a transition is obtained between a constant evaporation rate period (CRP) and a falling evaporation rate period (FRP). In Figure 4, the CRP corresponds to the initial linear evolution of *z*_1_(*t*) whereas the FRP corresponds to the period that follows when the absolute value of the slope  is smaller. For these experimental conditions in the CRP, the evaporation velocity determined from eq 1 with (*t*) = 0 (pinned meniscus) is 1.63 × 10^–6^ m/s, which is quite comparable to the experimental value  m/s obtained from a fit of the experimental data in the CRP. The inverse of the capillary number for this experiment is *Ca*^–1^ = 13.4, which gives *L* – *z*_2*t*_ = 4 mm. Thus, the CRP/FRP transition is expected when the water plug size is reduced by 4 mm, i.e., when *z*_1_ = *z*_1t_ = 6.8 mm. As can be seen from Figure 4, this prediction is in good agreement with the experimental data.

However, as can be seen from Figure 4, the model presented in the Supporting Information for determining the drying kinetics over both the CRP and the FRP leads to a faster drying. This discrepancy can be related to the prediction of the dry region size at the tube end during the FRP, i.e., the distance over which the evaporative meniscus recedes into the tube. Although the length  plotted in Figure 4 does not have exactly the same definition in the experiment (as shown in Figure 2a;  is the distance between the meniscus and the tube end measured from the images) and in the model where  is determined from eq 1 (see the Supporting Information), the model predicts a shorter receding distance. Since  is not large compared to the external mass transfer characteristic length δ, the evaporation rate is highly sensitive to the value of δ. Since the model depends on several physical and geometrical parameters, the question arises as to whether the discrepancy is due to questionable values of parameters or to questionable assumptions or approximations in the modeling. We have tested the model sensitivity to several physical parameters (liquid A viscosity and surface tension, contact angles, etc.). Increasing the PDMS contact angle to 35°, for instance, slightly improves the model prediction. Increasing the PDMS viscosity has also a favorable impact, but the discrepancy remains noticeable unless a significantly greater viscosity value than the one corresponding to the used PDMS is considered (i.e., μ_A_ = 98 Pa·s). Also, we have considered the situation where the water wettability is different in the tube end region compared to deeper inside the tube. The conclusion is that the most sensitive parameter is the tube diameter (again because the viscous pressure drop for a given velocity varies as *d*^–2^).

As illustrated in Figure 4a, considering a diameter of 250 μm, for instance, leads to a significantly better agreement between the model and the experimental data. Also as shown in Figure 4b, the agreement between the model and the experiment is quite good with regard to the evaporation rate (determined for the experiment using a simple finite difference from the experimental data, i.e., ). Figure 4b (inset) also shows the variation of the pressure in the gas plug as predicted by the model. The pressure decreases linearly from *P*_atm_ + *P*_cA_ to *P*_atm_ during the CRP and stays constant and equal to *P*_atm_ during the FRP. However, the tube diameter was measured on scanning electron microscopy (SEM) pictures and was found to be equal to the diameter specified by the supplier (300 μm). Therefore, further investigations are desirable to better understand why the model leads to a faster drying when setting the diameter of the tube to 300 μm, whereas the agreement is much better when a value of 250 μm is considered. Since the meniscus receding distance is small (900 μm, which is about three times the tube diameter in the experiment at the end of drying), i.e., not very large compared to the external mass transfer characteristic length (δ ≈ 180 μm), the details of the meniscus displacement in the tube end region matter. Thus, perhaps a more detailed investigation of the meniscus depinning in this region during the CRP/FRP transition could help explain the discrepancy with the experimental data. In any case, the model consistently predicts a drying kinetics in two main evaporation periods due to the viscous effects when *z*_1_(*t* = 0) > *Ca*^–1^*d*.

It is interesting to point out that the surface tension of liquid A was comparable or lower than the surface tension of the evaporating liquid (water) in the experiments. However, if liquid A has a larger surface tension than that of the evaporative liquid (for example, choosing water as fluid A and ethanol as the evaporative fluid where the surface tension of water is about three times larger than that of ethanol), one can imagine that the capillary action associated with the fluid A imbibition could lead to the rapid expulsion of the ethanol plug from the capillary. However, the exact situation also depends on the possible configuration of the evaporative liquid (ethanol in this discussion) meniscus at the exit of the tube. Due to the so-called capillary valve effect,^34^ a meniscus concave shape is possible depending on the wettability and tube geometry details in the tube end region. If the capillary valve effect is sufficient to block the imbibition, the evaporative liquid plug expulsion would not occur, and a slightly greater evaporation rate could be expected. The detailed analysis of this case (γ_A_ cos θ_A_ > γ_eva-liq_ cos θ_eva-liq_) is however beyond the scope of the present article.

Finally, the impact of fluid A properties on drying kinetics can be further illustrated by considering fluids which can undergo a phase transition from liquid to solid either by drying or by temperature change. This is notable the case of adhesives which are used in civil engineering to fix two materials together (tiles on a wall, for example). Moreover, glues have been frequently used in previous experiments to close capillaries and induce unidirectional drying.^16^ We have performed experiments with nail polish and epoxy glue which both solidify during their drying. As can be seen in Figure 5b, two different evaporation regimes can be observed for the entrapped water. During the first period, the meniscus of the entrapped water is pinned at the exit of the tube, and the fluid A, still liquid, enters the capillary from the other side. This regime is effectively described by eq 1 when setting (*t*) = 0. In those situations the evaporation rate is  mm^3^/s as described above. As time passes, fluid A solidifies and cannot advance in the capillary anymore. This phase transition leads to the second evaporation regime during which the air–water meniscus recedes inside the capillary. The transition between the two evaporative regimes is therefore a clear indication of the solidifying time of the glue used as a liquid plug to close the capillary.

## Conclusions

We have shown that the dynamics of unidirectional drying of water in the presence of an entrapped gas plug in a single capillary are strongly dependent on whether the end of the capillary is connected to a liquid reservoir or clogged with a solid material. Contrary to the well-known case of the Stefan tube drying, evaporation can lead to capillary pumping when the round capillary is connected to a liquid reservoir and a much faster constant-rate evaporation. In this configuration, the drying regime is not affected by the geometry of the pore, i.e., the shape of the tube cross section. If the liquid closing the capillary is sufficiently viscous, a transition from a constant-rate evaporation regime to a diffusive regime can also be observed when the tube diameter and the size of the entrapped gas plug are sufficiently small. Finally, we studied the influence of solidifying glues on the drying of capillaries partially filled with water. The use of such glues to seal capillaries can lead to drying behavior with two periods: a first period where the drying rate is constant and some glue enters the capillary, followed by a second diffusive regime once the glue is solidified. Such behaviors remain relevant in situations where soils or porous materials in general are connected to a liquid reservoir or materials which can solidify (or gelify) over time.

## Supporting Information Available

Results of the experiments with square capillaries and more details on the determination of δ; details of the model about the spontaneous imbibition step in a capillary closed by a fluid and corresponding calculations and details of the model used in Figure 4 (drying with viscous effect) (PDF)

## References

[ref1] Or D.; Lehmann P.; Shahraeeni E.; Shokri N. Advances in Soil Evaporation Physics—A Review. Vadose Zone J. 2013, 12, vzj2012.0163 10.2136/vzj2012.0163.

[ref2] Mujumdar A. S., Ed. Handbook of Industrial Drying, 4th ed.; CRC Press: Boca Raton, FL, 2014.

[ref3] Wu R.; Prat M. Mass Transfer Driven Evaporation from Capillary Porous Media; CRC Press: Boca Raton, FL, 2022.

[ref4] Shokri N.; Lehmann P.; Or D. Critical evaluation of enhancement factors for vapor transport through unsaturated porous media. Water Resour. Res. 2009, 45. 10.1029/2009WR007769.

[ref5] Olbricht W. L. Pore-Scale Prototypes of Multiphase Flow in Porous Media. Annu. Rev. Fluid. Mech 1996, 28, 187–213. 10.1146/annurev.fl.28.010196.001155.

[ref6] Zhao B.; et al. Comprehensive comparison of pore-scale models for multiphase flow in porous media. Proc. Natl. Acad. Sci. U.S.A 2019, 116, 13799–13806. 10.1073/pnas.1901619116. 31227608 PMC6628826

[ref7] Blunt M. J. Flow in porous media — pore-network models and multiphase flow. Curr. Opin. Colloid Interface Sci. 2001, 6, 197–207. 10.1016/S1359-0294(01)00084-X.

[ref8] Prat M. Recent advances in pore-scale models for drying of porous media. Chem. Eng. Sci. 2002, 86, 153–164. 10.1016/S1385-8947(01)00283-2.

[ref9] Qin F.; Zhao J.; Kang Q.; Derome D.; Carmeliet J. Lattice Boltzmann Modeling of Drying of Porous Media Considering Contact Angle Hysteresis. Transp. Porous Media 2021, 140, 395–420. 10.1007/s11242-021-01644-9. 34720284 PMC8550062

[ref10] Stefan J. The Dynamics of Capillary Flow. Sitzungsber. Akad. Wiss. Wien 1871, 63.

[ref11] Mitrovic J. Josef Stefan and his evaporation–diffusion tube—the Stefan diffusion problem. Chem. Eng. Sci. 2012, 75, 279–281. 10.1016/j.ces.2012.03.034.

[ref12] Camassel B.; Sghaier N.; Prat M.; Ben Nasrallah S. Evaporation in a capillary tube of square cross-section: application to ion transport. Chem. Eng. Sci. 2005, 60, 815–826. 10.1016/j.ces.2004.09.044.

[ref13] Shahidzadeh-Bonn N.; Rafai S.; Bonn D.; Wegdam G. Salt Crystallization during Evaporation: Impact of Interfacial Properties. Langmuir 2008, 24, 8599–8605. 10.1021/la8005629. 18652495

[ref14] Wang S.; Zhou H.; Sun Z.; Xu S.; Ouyang W.; Wang L. Evolution of concentration and phase structure of colloidal suspensions in a two-ends-open tube during drying process. Sci. Rep 2020, 10, 9084 10.1038/s41598-020-65879-0. 32493983 PMC7270103

[ref15] Chauvet F.; Duru P.; Geoffroy S.; Prat M. Three Periods of Drying of a Single Square Capillary Tube. Phys. Rev. Lett. 2009, 103, 124502 10.1103/PhysRevLett.103.124502. 19792442

[ref16] Keita E.; Koehler S. A.; Faure P.; Weitz D. A.; Coussot P. Drying kinetics driven by the shape of the air/water interface in a capillary channel. Eur. Phys. J. E 2016, 39, 23 10.1140/epje/i2016-16023-8. 26920526

[ref17] Wu R.; Zhang T.; Ye C.; Zhao C. Y.; Tsotsas E.; Kharaghani A. Pore network model of evaporation in porous media with continuous and discontinuous corner films. Phys. Rev. Fluids 2020, 5, 014307 10.1103/PhysRevFluids.5.014307.

[ref18] Yiotis A. G.; Boudouvis A. G.; Stubos A. K.; Tsimpanogiannis I. N.; Yortsos Y. C. Effect of liquid films on the drying of porous media. AIChE J. 2004, 50, 2721–2737. 10.1002/aic.10265. 14524931

[ref19] Prat M. On the influence of pore shape, contact angle and film flows on drying of capillary porous media. Int. J. Heat Mass Transfer 2007, 50, 1455–1468. 10.1016/j.ijheatmasstransfer.2006.09.001.

[ref20] Wijnhorst R.; de Goede T. C.; Bonn D.; Shahidzadeh N. Surfactant Effects on the Dynamics of Capillary Rise and Finger Formation in Square Capillaries. Langmuir 2020, 36, 13784–13792. 10.1021/acs.langmuir.0c01965. 33164529

[ref21] Dufresne E. R.; Corwin E. I.; Greenblatt N. A.; Ashmore J.; Wang D. Y.; Dinsmore A. D.; Cheng J. X.; Xie X. S.; Hutchinson J. W.; Weitz D. A. Flow and Fracture in Drying Nanoparticle Suspensions. Phys. Rev. Lett. 2003, 91, 224501 10.1103/PhysRevLett.91.224501. 14683242

[ref22] Lidon P.; Salmon J.-B. Dynamics of unidirectional drying of colloidal dispersions. Soft Matter 2014, 10, 4151–4161. 10.1039/c3sm52528g. 24756218

[ref23] Van Brakel J.; Mujumdar A. S. Mass Transfer in Convective Drying; Hemisphere Publishing Corporation: Washington, DC, 1980.

[ref24] Coussot P. Scaling approach of the convective drying of a porous medium. Eur. Phys. J. B 2000, 15, 557–566. 10.1007/s100510051160.

[ref25] Parikh D. M. Vacuum Drying: Basics and Application. Chem. Eng. 2015, 122, 48–54.

[ref26] Diouf B.; Geoffroy S.; Chakra A. A.; Prat M. Locus of first crystals on the evaporative surface of a vertically textured porous medium. Eur. Phys. J. Appl. Phys. 2018, 81, 11102 10.1051/epjap/2018170340.

[ref27] Bird R. B.; Stewart W. E.; Lightfoot E. N. Transport phenomena; John Wiley and Sons, Inc.: New York, 1961.

[ref28] Lee C. Y.; Wilke C. R. Measurements of Vapor Diffusion Coefficient. Ind. Eng. Chem. 1954, 46, 2381–2387. 10.1021/ie50539a046.

[ref29] Chauvet F.; Duru P.; Prat M. Depinning of evaporating liquid films in square capillary tubes: Influence of corners’ roundedness. Phys. Fluids 2010, 22, 112113 10.1063/1.3503925.

[ref30] Washburn E. W. The Dynamics of Capillary Flow. Phys. Rev. 1921, 17, 273–283. 10.1103/PhysRev.17.273.

[ref31] Fazio R.; Iacono S. An analytical and numerical study of liquid dynamics in a one-dimensional capillary under entrapped gas action. Math. Methods Appl. Sci. 2014, 37, 2923–2933. 10.1002/mma.3030.

[ref32] de Gennes P.-G.; Brochard-Wyart F.; Quéré D. Capillarity and Wetting Phenomena: drops, bubbles, pearls, waves; Springer Science Business Media: New York, 2003.

[ref33] Redon C.; Brzoska J. B.; Brochard-Wyart F. Dewetting and slippage of microscopic polymer films. Macromolecules 1994, 27, 468–471. 10.1021/ma00080a021.

[ref34] Wu R.; Kharaghani A.; Tsotsas E. Capillary valve effect during slow drying of porous media. Int. J. Heat Mass Transf 2016, 94, 81–86. 10.1016/j.ijheatmasstransfer.2015.11.004.

