# Supplementary material for: Is Unidirectional Drying in a Round Capillary Always Diffusive?
Source: Langmuir. 2023 Apr 6;39(15):5462–8. doi: 10.1021/acs.langmuir.3c00169 (PMC10116593; doi:10.1021/acs.langmuir.3c00169)
Supplement: Supplementary file 1 — la3c00169_si_001.pdf [file la3c00169_si_001.pdf]

Is unidirectional drying in a round capillary always diffusive?

Romane Le Dizès Castell,[*,†] Marc Prat,[‡] Sara Jabbari Farouji,[†] and Noushine Shahidzadeh[†]

† *Van der Waals - Zeeman Institute, University of Amsterdam, The Netherlands*

‡ *Institut de Mécanique des Fluides de Toulouse (IMFT), Université de Toulouse, France*

E-mail: r.ledizes2@uva.nl

Supporting Information

The Supporting Information contains details on:

1. Results of the drying experiments in the square capillaries,
2. Determination of δ in configuration (b) for round capillaries,
3. Spontaneous imbibition step of fluid A in the capillary in configuration (b)
4. Model about the drying with viscous effect in configuration (b)

1. Results in square capillaries Fig. 1 shows the results for configuration (a) and configuration (b) with square capillaries.

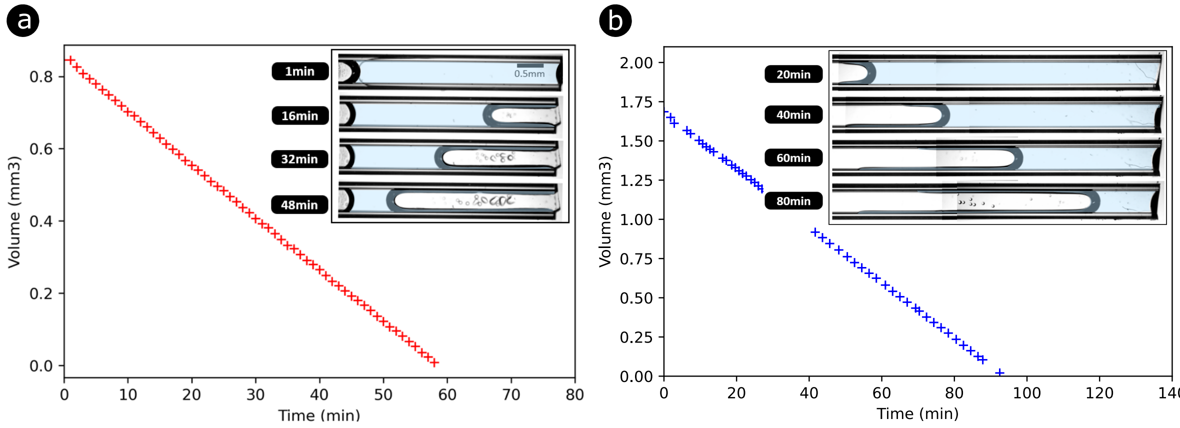

Figure 1: Drying of water (highlighted in blue) in square capillaries melted on one side (a) ($\frac{\mathrm{d}V}{\mathrm{d}t} = 1.46 * 10^{-2}$ mm^3/min) or closed by a water droplet (b) ($\frac{\mathrm{d}V}{\mathrm{d}t} = 1.8 * 10^{-2}$ mm^3/min).

In situation (a) where the capillary is closed by a melted plug, the evaporative meniscus recedes in the capillary and some corner flows are forming on the side of the capillary as described in the litterature.[1–4] This leads to a constant rate evaporation due to the presence of corner flows. In situation (b), as for round capillaries, the meniscus remains pinned at the evaporative side and one CRP is also observed until the end of the drying. Thus, performing the experiments with different geometries (round or square) does not affect the evaporation regimes. We can nevertheless notice that the evaporation rate is higher for situation (b) ($1.8 * 10^{-2}$ mm^3/min) than for situation (a) ($1.46 * 10^{-2}$ mm^3/min). This can be explained by the fact that the evaporation surface is higher in situation (b) compared to situation (a) where water evaporates mainly at the tip of the fingers.[2,5]

2. Determination of δ in configuration (b) In configuration (b) in Fig. 2, the volume of the water plug drops linearly with time and the evaporation rate is constant. As the water evaporates at the entrance of the capillary during the whole drying, it is possible to

determine the value of the external diffusive length δ using Fick's law.

$$\frac{\mathrm{d}m}{\mathrm{d}t} = AD_v \frac{M_v}{RT} p_{vsat} \frac{1 - RH}{\delta} \tag{1}$$

The diffusive length is extracted from every experiment and is equal to $\delta = 195 \pm 12$ μm which is in the same range as in configuration (a).The slightly smaller value found here can be qualitatively explained by the fact that the external transfer is slightly different when the meniscus is right at the entrance of the tube and when the meniscus in deeper inside the tube due to the difference in the structure of the local vapor partial pressure distribution in the tube exit region (the local evaporation flux is expected to be more uniform over the tube entrance when the meniscus is inside the tube compared to the case of a meniscus located right at the tube entrance. In the latter case, a greater local flux at the tube end periphery is expected, somewhat similarly to the local evaporation flux distribution over an evaporative flat droplet). Nevertheless, these considerations would deserve to be explored in more details from numerical simulations, in the spirit of the results shown in[6] , where the evaporation rate from a flat meniscus at a channel end is compared to the one obtained when the meniscus has partially receded into the channel.

3. Spontaneous imbibition step In configuration (b), the very fast spontaneous imbibition period occurring when the capillary is connected to the droplet of fluid A can be analyzed using a classical Washburn model combined with the consideration of the pressure build-up in the gas plug and inertial effects in the liquid.[7,8] The equation governing the position of fluid A meniscus can be expressed as:[8]

$$\rho_A (\frac{\mathrm{d}\ell_A}{\mathrm{d}t})^2 = P_{cA} - \frac{32\mu_A}{d^2} \ell_A \frac{\mathrm{d}\ell_A}{\mathrm{d}t} + P_{atm}(1 - \frac{L - z_1}{L - z_1 - \ell_A}) \tag{2}$$

where $\ell_A = L - z_2$ is the position of fluid A meniscus measured from the capillary left end (Fig. 1 in the main text), ρ_A is fluid A density and $P_{cA} = \frac{4\gamma_A cos\theta_A}{d}$. The properties for the

three liquids considered are given in Table 1.

Table 1: Physical properties of water, glycerol and PDMS used in the computations

	ρ_A (k/m^3)	γ_A (N/m)	θ_A (°)	μ_A (Pa.s)
water	1000	$72 * 10^{-3}$	40	10^{-3}
Glycerol	1260	63.4	10	1.5
PDMS[9]	965	20	16	98

Solving numerically Eq. 4 (in the main text), for $L = 19.4$ mm, $z_1 = 5$ mm leads to the results shown in Fig. 2. As can be seen, the greater the viscosity, the longer is the imbibition period (1000 times longer with PDMS compared to water for instance). Nevertheless, the imbibition period is very short, less than 1 second, even for the most viscous fluid tested. Interestingly, the forces controlling the spontaneous imbibition differs depending on the fluid. For water, the less viscous fluid, the imbibition is dominated by the capillary and inertial forces (the viscous term $\frac{32\mu_A}{d^2}\ell_A\frac{\mathrm{d}\ell_A}{\mathrm{dt}}$ can be neglected in Eq. 2) except at the very end when the meniscus velocity approaches zero). By contrast, the inertial effects (*i.e.* the term $\rho_A(\frac{\mathrm{d}\ell_A}{\mathrm{dt}})^2$ in Eq. 2) are negligible for the most viscous fluid (PDMS). The imbibition is controlled by the capillary and viscous effects. The situation for the third fluid, glycerol, is intermediate. Inertial effects are dominant in a first period and the viscous effects become gradually more and more important as the hydrostatic equilibrium is approached.

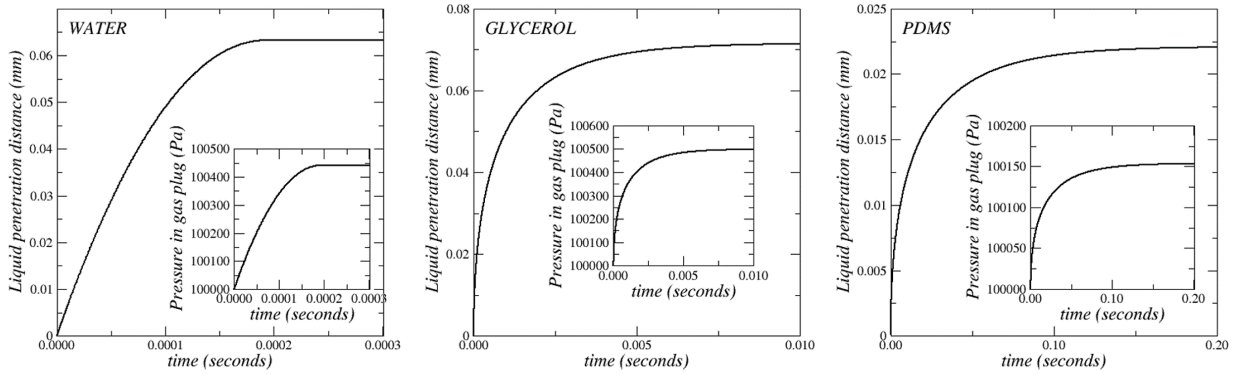

Figure 2: Evolution of fluid A penetration distance ℓ_A during the period of spontaneous imbibition for the three fluids. The insets show the pressure evolution in the gas plug.

The penetration distance is shorter for the most viscous fluid but is quite short for the

three fluids, on the order of a few tens of micrometers. The pressure in the gas plug is computed applying the ideal gas law as $P_g(t) = P_{atm}\frac{L-z_1(t=0)}{L-z_1(t=0)-\ell_A(t)}$. As discussed in the main text, the maximum pressure in the gas plug (which corresponds to the plateaus in Fig. 2) is given by $P_g \approx P_{atm} + P_{cA}$, neglecting the slight overpressure due to the height of the liquid in the droplet (see main text).

4. Drying with viscous effect (configuration (b) in Fig. 1 in the main text) The drying rate is expressed using the following equation:

$$\frac{\mathrm{d}m}{\mathrm{d}t} = AD_v\frac{M_v}{RT}p_{vsat}\frac{1-RH}{\delta+\ell(t)} \tag{3}$$

Neglecting the viscous pressure drop in the liquid water plug and the gas plug and considering the viscous pressure drop in the region of the tube occupied by fluid A, the pressure in the water right behind the evaporative meniscus can be expressed as:

$$P_w = P_{atm} - \Delta P_A + P_{cA} - P_{cw} \tag{4}$$

Where $P_{cA} = \frac{4\gamma_A cos\theta_A}{d}$, $P_{cw} = \frac{4\gamma_w cos\theta_w}{d}$, $\Delta P_A = \frac{32\mu_A}{d^2 v_{ev}}(L - z_2)$ and v_{ev} is the evaporation velocity. The pressure jump P_{cem} at the evaporative meniscus is then given by,

$$P_{cem} = P_{atm} - P_w = \Delta P_A - P_{cA} + P_{cw} \tag{5}$$

The evaporative meniscus is pinned at the tube end as long as $P_{cem} < P_{cw}$. This corresponds to a first period where the evaporation rate is constant. The evolution of z_2 in this period is given by $\frac{dz_2}{dt} = \frac{dz_1}{dt} = -v_{ev}$. As fluid A penetrates further in the tube, *i.e.* as the penetration distance $(L - z_2)$ increases, the viscous pressure drop increases until $P_{cem} = P_{cw}$. According to Eq. 5, this corresponds to $(L - z_{2t})$ given by solving the equation $\Delta P_A – P_{cA} = 0$. Thus $z_{2t} = L - \frac{d^2}{32\mu_A v_{ev}}\frac{4\gamma_A cos\theta_A}{d}$ or $z_{2t} = L - Ca^{-1}d$ with $Ca^{-1} = \frac{4\gamma_A cos\theta_A}{32\mu_A v_{ev}}$. When this situation

is reached, the evaporative meniscus starts receding into the tube. In the second period, the pressure in the gas plug is imposed by the curvature of the water menisci in the tube. Therefore, $P_g = P_{atm}$. As a result, the viscous volumetric flow rate q_A in fluid A is given by:

$$q_A = -A\frac{\mathrm{d}z_2}{\mathrm{d}t} = A\frac{d^2}{32\mu_A}\frac{P_{cA}}{L - z_2} \tag{6}$$

This equation gives the position of fluid A meniscus as a function of time in the second period, namely:

$$z_2(t) = L - \sqrt{(L - z_{2t})^2 + \frac{2d^2 P_{cA}}{32\mu_A}(t - t_t)}, \qquad (t \geq t_t) \tag{7}$$

where t_t is the time marking the transition between the constant evaporation rate period and the falling evaporation rate period. This time is given by the equation$(L - z_{2t} = v_{ev}t_t)$ where $v_{ev} = \frac{1}{\rho_w}D_v\frac{M_v}{RT}p_{vsat}\frac{(1-RH)}{\delta}$ according to Eq. 3.

We have still two unknowns to be determined during the second period: $z_1(t)$ and $\ell(t)$ where $\ell(t)$ is the position of the evaporative meniscus in the tube. Expressing that the evaporation corresponds the water mass loss of the water plug yields:

$$\frac{\mathrm{d}(z_1 - \ell)}{\mathrm{d}t} = \frac{1}{\rho_w A}\frac{\mathrm{d}m}{\mathrm{d}t} = \frac{1}{\rho_w}D_v\frac{M_v}{RT}p_{vsat}\frac{1 - RH}{\delta + \ell(t)} \tag{8}$$

In the considered experiments, the pressure variation in the gas plug remains small compared to the atmospheric pressure. As the result, the gas plug length variations are small. An approximation consists in neglecting these variations, which allows to compute $z_1(t)$ from the relationship,

$$z_1(t) = z_2(t) - L + z_1(t = 0) \tag{9}$$

noting that the initial imbibition distance is very small, *i.e.* $z_2(t) \approx L$. The method of solution then consists in determining $z_2(t)$ for the next time step from Eq. 7. Then $z_1(t)$ is determined from Eq. 9 whereas ℓ is computed from Eq. 8 expressed as $-\frac{\mathrm{d}\ell}{\mathrm{d}t} =$

$\frac{1}{\rho_w} D_v \frac{M_v}{RT} p_{vsat} \frac{1-RH}{\delta+\ell(t)} - \frac{\mathrm{d}z_1}{\mathrm{d}t}$ using a simple Euler scheme.

References

(1) Prat, M. On the influence of pore shape, contact angle and film flows on drying of capillary porous media. *Int. J. Heat Mass Transfer* **2007**, *50*, 1455–1468.

(2) Chauvet, F.; Duru, P.; Geoffroy, S.; Prat, M. Three Periods of Drying of a Single Square Capillary Tube. *Phys. Rev. Lett.* **2009**, *103*, 124502.

(3) Chauvet, F.; Duru, P.; Prat, M. Depinning of evaporating liquid films in square capillary tubes: Influence of corners' roundedness. *Phys. Fluids* **2010**, *22*, 112–113.

(4) Yiotis, A. G.; Boudouvis, A. G.; Stubos, A. K.; Tsimpanogiannis, I. N.; Yortsos, Y. C. Effect of liquid films on the drying of porous media. *AIChE J.* **2004**, *50*, 2721–2737.

(5) Prat, M. Recent advances in pore-scale models for drying of porous media. *Chem. Eng. Sci.* **2002**, *86*, 153–164.

(6) Ghiringhelli, E.; Marcoux, M.; Geoffroy, S.; Prat, M. Evaporation in a single channel in the presence of particles. *Colloids Surf. A Physicochem. Eng. Asp.* **2023**, *656*, 130432.

(7) Quéré, D. Inertial capillarity. *Europhys. Lett.* **1997**, *39*, 533.

(8) Fazio, R.; Iacono, S. An analytical and numerical study of liquid dynamics in a one-dimensional capillary under entrapped gas action. *Math. Methods Appl. Sci.* **2014**, *37*, 2923–2933.

(9) Redon, C.; Brzoska, J. B.; Brochard-Wyart, F. Dewetting and slippage of microscopic polymer films. *Macromolecules* **1994**, *27*, 468–471.
